# A stretchable frequency reconfigurable antenna controlled by compressive buckling for W-band applications

**DOI:** 10.1038/s41378-025-00890-x

**Published:** 2025-05-13

**Authors:** Qi Wang, Zetian Wang, Yang Yang, Chi Zhang, Mengdi Han, Wei Wang, Yufeng Jin

**Affiliations:** 1 Peking University Shenzhen Graduate School, Peking University, Shenzhen, China; 2 School of Integrated Circuits, Peking University, Beijing, China; 3 National Key Laboratory of Advanced Micro and Nano Manufacture Technology, Beijing, China; 4 Beijing Advanced Innovation Center for Integrated Circuits, Beijing, China; 5 School of Future Technology, Peking University, Beijing, China

**Keywords:** Electrical and electronic engineering, Electronic devices

## Abstract

Reconfigurable antennas have attracted significant interest because of their ability to dynamically adjust radiation properties, such as operating frequencies, thereby managing the congested frequency spectrum efficiently and minimizing crosstalk. However, existing approaches utilizing switches or advanced materials are limited by their discrete tunability, high static power consumption, or material degradation for long-term usage. In this study, we present a W-band frequency reconfigurable antenna that undergoes a geometric transformation from a two-dimensional (2D) precursor, selectively bonded to a prestretched elastomeric substrate, into a desired 3D layout through controlled compressive buckling. Modeling the buckling process using combined mechanics-electromagnetic finite element analysis (FEA) allows for the rational design of the antenna with desired strains applied to the substrate. By releasing the substrate at varying compression ratios, the antenna reshapes into different 3D configurations, enabling continuous frequency reconfigurability. Simulation and experimental results demonstrate that the antenna’s resonant frequency can be tuned from 77 GHz in its 2D state to 94 GHz in its 3D state in a folded-dipole-like design.

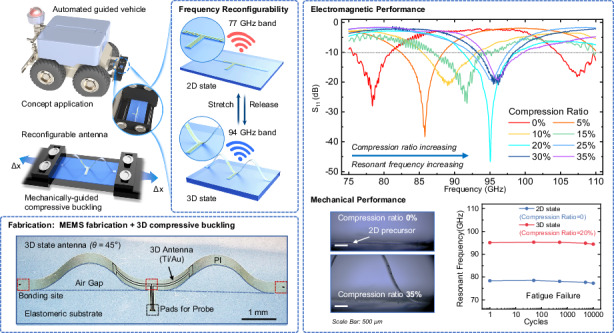

## Introduction

Reconfigurable antennas have demonstrated versatile applicability because they can adjust the resonant frequency, radiation pattern, or polarization, enabling diverse target functions via strategic physical modifications. With radio frequency (RF) reconfigurability, these antennas can operate across a broad range of frequencies, enhancing bandwidth performance in compact and multifunctional RF systems^1–7^.

Frequency reconfigurability has been achieved through various methods, including modifying the antenna’s geometric configuration or dielectric properties via electrical switches, advanced materials, or mechanical deformations. Electrical approaches commonly employ miniaturized switchable components, such as PIN diodes^8–11^, varactors^12–15^, and RF MEMS switches^16–18^, to connect radiating elements and alter resonant characteristics. However, the bias circuits required for these switchable components introduce parasitic effects and contribute to static power consumption. Moreover, switchable components like PIN diodes and RF MEMS switches typically provide discrete reconfigurability with limited states, restricting continuous tuning capabilities. Additionally, in the millimeter wave (mm-wave) spectrum, tuning components are often electrically large, which significantly affects the radiated field. Advanced materials, such as memory alloys^19–21^, polymers^22–24^, phase-change materials^25^, and liquid metals^26^, can actively alter dielectric properties or deform to adjust the operating frequency. These materials, stimulated by light^27^, heat^28^, or electric and magnetic fields, largely avoid the drawbacks associated with bias circuits and enable continuous tunability. However, external stimuli under specific environmental conditions can degrade the system’s wireless performance. Mechanically-guided methods have been investigated to enable controlled and reversible deformations while mitigating material degradation caused by external electromagnetic or thermal fields. Consequently, the mechanically-guided approach offers a versatile solution suitable for various operating scenarios, including harsh environmental conditions such as extreme temperatures and electromagnetic interference, thereby ensuring robust and reliable performance across a broad spectrum of dynamic and challenging operational environments.

The mechanically guided method leverages the geometric transformation of a 2D precursor into a 3D configuration via compressive buckling, enabling antenna reconfigurability^29,30^. In this approach, a patterned 2D precursor is selectively bonded to a prestrained substrate, and releasing the prestrain initiates a predictable and reliable 2D-to-3D transition. This method eliminates the need for high temperatures, electromagnetic stimuli, or bias circuits^31–33^, and is compatible with various materials, including semiconductors, metals, and polymers, and is scalable from meter-length dimensions down to the sub-micrometer scale. Additionally, the use of flexible materials also allows conformity to complex curvilinear surfaces, and the buckling process extends design flexibility by expanding accessible topologies into 3D space^34,35^. Previous studies have demonstrated the potential of this method, exemplified by a 3D stretchable dipole antenna for wearable applications^36^ and a radiation-pattern-reconfigurable antenna for complex surfaces^37^. However, these designs have not achieved frequency reconfigurability, as they typically operate at lower frequencies (e.g., Wi-Fi bands at 2.45 GHz and 5 GHz), where large wavelengths of the antennas mean that deformations cause almost few frequency shifts. In contrast, mechanically-guided methods show significant promise for designing frequency reconfigurable antennas in the mm-wave spectrum (30–300 GHz), where even slight dimensional changes can induce noticeable frequency shifts. This frequency range is critical for next-generation technologies, offering distinct advantages for imaging and sensing in harsh environments such as smoke, dust, fog, rain, and snow, where optical systems like cameras and LiDARs face severe limitations^38,39^. Frequency reconfigurable antennas in the mm-wave band enable compact, multi-band, and multifunctional solutions, addressing the demands of diverse applications, such as automotive radar and advanced logistics systems^40–42^. For instance, in automotive systems, frequency reconfigurability is essential for switching between the 77 GHz band, which supports adaptive cruise control and collision avoidance, and the 94 GHz band, which facilitates precise object detection and high-resolution imaging for advanced driver assistance systems (ADAS)^43,44^.

In this study, we report the design and fabrication of a stretchable, frequency reconfigurable antenna operating in the W-band (75–110 GHz) based on a mechanically-guided approach. This method employs advanced microfabrication technologies to create 2D precursor structures selectively bonded to prestretched substrates. By releasing the prestrain, the antenna undergoes a controlled 2D-to-3D transformation via compressive buckling, ensuring reliable and repeatable reconfigurability. Comprehensive theoretical and experimental assessments, supported by electromagnetic and mechanical simulations, validate the antenna’s tunability. The antenna achieves tunability by buckling the dipole strip, forming a tunable air gap between the 3D antenna and the substrate. This design enables active control of the resonant frequency within a continuous range from 77 GHz to 94 GHz, offering significant potential for integration into platforms that require compact, multi-band, and adaptable antennas. Specifically, the proposed antenna is ideal for logistics robots, unmanned aerial vehicles (UAVs), and automated guided vehicles (AGVs). At 77 GHz, it supports short-range radar sensing for obstacle detection and dynamic path planning, ensuring efficient navigation within warehouses and distribution centers. At 94 GHz, it facilitates high-resolution imaging for precise object tracking and real-time operations such as sorting, loading, and unloading. These capabilities make the antenna a promising solution for automotive, industrial, and logistics applications, where multifunctionality, low payload weight, and compactness are critical.

## Results

### Device structure and working mechanism

**Fig. 1:**
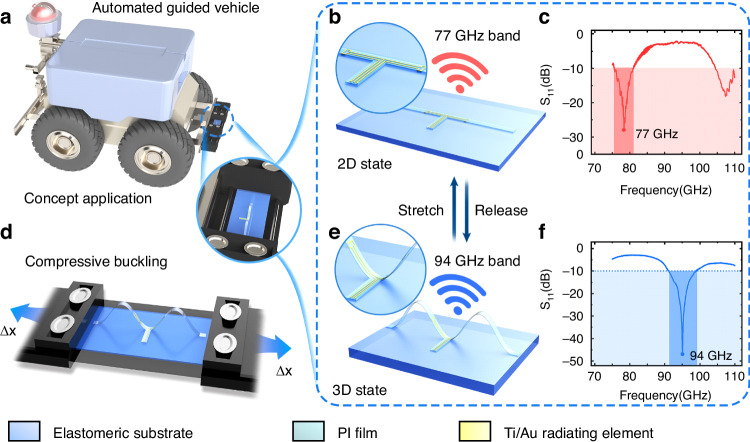
Schematic illustrations of the compressive buckling reconfigurable antenna. **a** The operating concept of the reconfigurable antenna for automotive platforms and automated guided vehicles (AGVs). **b** Schematic illustration of the 2D precursor bonded onto a prestrained elastomeric substrate. **c** The reflection coefficient (S_11_) characteristics of the antenna in its 2D state exhibit a resonant frequency of 77 GHz. **d** Configuration image of the stretchable antenna following strain release. **e** Schematic illustration of the buckling-guided process used to configure a reconfigurable 3D antenna by releasing and stretching the antenna to achieve a predefined geometric configuration at a specified compression ratio. **f** S_11_ characteristics of the antenna in its 3D state, demonstrating a resonant frequency of 94 GHz

Figure 1 illustrates the schematic diagrams of the proposed frequency reconfigurable antenna based on the mechanically-guided method. The 2D precursor design comprises a polyimide (PI) film ribbon, incorporating a titanium/gold (Ti/Au) radiating element layer sandwiched within. The flexible ribbon is selectively bonded onto the prestrained elastomeric substrate (Dragon Skin, Smooth-On) at three predetermined sites. Upon releasing the prestrain, the resulting compressive forces induce lateral buckling in the 2D precursor structure. As shown in Fig. 1d, this process results in a 3D configuration of the PI ribbon structure. The radiating element, designed in a folded-dipole shape, achieves frequency tunability from 77 GHz to 94 GHz through mechanical deformation induced by releasing the elastomeric substrate. The antenna can be reshaped reversibly by repeatedly stretching and releasing the elastomeric substrate between the 2D and 3D configurations, enabling both geometric reconfigurability and tunability in operating frequency. The mechanically-guided reconfigurable approach allows real-time tuning without requiring switchable components or external stimuli such as light, heat, or electromagnetic fields, enabling dynamic adaptation to varied tasks, including localization in the 77 GHz band and high-resolution imaging in the 94 GHz band. Additionally, the flexible design conforms to complex surfaces, thereby optimizing space utilization while ensuring robust electromagnetic performance.

### Design of the antenna

**Fig. 2:**
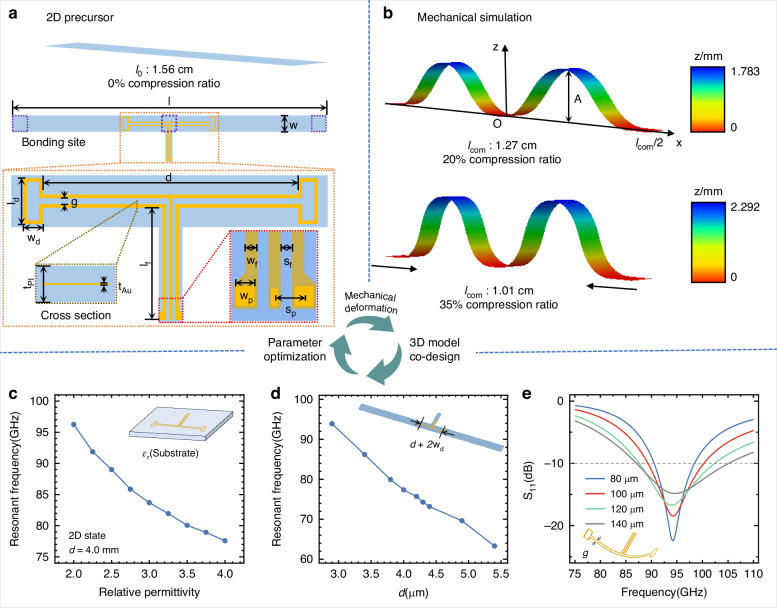
Antenna design and simulation results. **a** Schematic of the 2D precursor design for the antenna. **b** 3D configurations of the antenna, as obtained from mechanical FEA simulations at compression ratios of 20% and 35%, with color gradients indicating out-of-plane displacement along the z-axis. **c** Simulation results demonstrate that an increase in the relative permittivity of the elastomeric substrate leads to a decrease in the resonant frequency of the antenna when the antenna length is held constant. **d** Simulation results show that increasing the proposed antenna length (*d*) decreases the resonant frequency within both the Dragon Skin substrate and the PI film. **e** S_11_ characteristics for varying gaps (*g*) between the upper and lower radiating elements

Figure 2 outlines the procedures for synthesizing the reconfigurable antenna. Electromagnetic and mechanics simulations are conducted to evaluate the electromagnetic performance and predict the 3D profile of the antenna under varying mechanical compression ratios. The 2D precursor is modeled and simulated in the ANSYS HFSS electromagnetic simulator. Figure 2 presents a simulation model along with the dimensional parameters of the reconfigurable antenna’s 2D precursor, consisting of a PI supporting layer sandwiching a metallic layer. The dimensional parameters obtained from the electromagnetic simulations are imported into ABAQUS, a commercial software for mechanical finite element analysis (FEA), to predict antenna deformation under varying levels of mechanical compression, as shown in Fig. 2b. The resulting 3D configurations are iteratively refined through additional electromagnetic simulations. Further design and optimization details are provided in the “Materials and Methods” section.

A folded-dipole antenna is designed for an mm-Wave reconfigurable system. Unlike traditional dipole antennas, this design significantly enhances antenna gain by increasing the area of the radiating elements and achieves a broader operating bandwidth that supports various communication protocols and standards. The geometrical reconfiguration of the radiating elements is crucial for the antenna’s operating frequency. Specifically, the length of the dipole antenna (*L*) is inversely proportional to the antenna’s operating frequency^45^:

1 $${f}_{c}=c/(2L\sqrt{{\varepsilon }_{r}})$$

where *f*_c_ is the resonant frequency of the dipole antenna, *ε*_r_ is the relative permittivity of the medium material, and *c* is the velocity of light. As the antenna deforms from the 2D to the 3D state, the *ε*_r_ gradually decreases due to the formation of air gaps between the radiating elements and the elastomeric substrate. For the constant length of the folded-dipole antenna (*w*_d_ + *d*/2) in Fig. 2a, the reduced *ε*_r_ leads to a higher operating frequency, as illustrated in Figs. 2c, S3a. The effective relative permittivity of the air gap and elastomeric substrate is related to the height of the 3D buckling antenna^46^:

2 $${\varepsilon }_{{\rm{r}},{\rm{eff}}}=\left(\frac{{h}_{{\rm{air}}}+{{\rm{t}}}_{{\rm{ela}}}}{\frac{{h}_{{\rm{air}}}}{{\varepsilon }_{{\rm{r}},{\rm{air}}}}+\frac{{{\rm{t}}}_{{\rm{ela}}}}{{\varepsilon }_{{\rm{r}},{\rm{ela}}}}}+{\varepsilon }_{{\rm{r}},{\rm{ela}}}\right)/2$$

3 $${h}_{{\rm{air}}}=({w}_{{\rm{d}}}+d/2)\cdot \,\cos \theta$$

where *ε*_r,eff_ is the effective relative permittivity of the air gap and substrate, *h*_air_ is the height of the air gap, *t*_ela_ is the thickness of the elastomeric substrate, and *ε*_r,air_ and *ε*_r,eff_ are the relative permittivities of the air and the elastomeric substrate, respectively, and *θ* is the angle between the center of the antenna and the elastomeric substrate (*θ* = 0° when the antenna is flat, and *θ* = 90° when the antenna is perpendicular to the substrate). From Eqs. (2) and (3), it is evident that as the *θ* increases, the *ε*_r,eff_ decreases. However, as the antenna buckles to a greater height (larger *θ*), the rate of decrease in *ε*_r,eff_ slows, leading to a slight increase in the operating frequency. In other words, when the compression ratio of the substrate reaches a certain threshold, the deformation of the antenna becomes minimal, allowing the operating characteristics to remain stable under this prestrain. Furthermore, the low-loss air gap between the antenna and substrate reduces the dielectric loss and enhances the antenna’s operating bandwidth. Therefore, the stability of the 3D antenna’s operation has been significantly enhanced using mechanically-guided method.

For the specified dielectric materials, PI and Dragon Skin, the antenna length (*d*, as shown in Fig. 2a) is a critical design parameter because it directly influences the antenna’s resonant frequency. As shown in Figs. 2d, S3b, an increase in *d* results in a corresponding decrease in the resonant frequency. Additionally, a coplanar waveguide (CPW) configuration is employed as the feed line due to its favorable characteristics in terms of impedance matching and ease of integration within compact designs. To ensure optimal performance, simulations are conducted on CPW feed lines featuring a line width (*w*_f_) of 50 μm and line spacing (*s*_f_) of 50 μm using the SI 9000 impedance calculator; these dimensions are critical for achieving an input impedance of 50 Ω. This CPW structure is designed to ensure direct interconnection with the chip’s pad, facilitating effective impedance matching while minimizing radiation loss during transmission—a crucial consideration for high-frequency signals. The 3D configurations obtained from ABAQUS simulations are subsequently analyzed in HFSS to evaluate and optimize the electromagnetic performance of the antenna in its 3D state. The gap between the upper and lower radiating element parts (*g*, as shown in Fig. 2a) is a key parameter that significantly influences impedance matching and bandwidth in the 3D state. As shown in Fig. 2e, reducing the value of *g* decreases the reflection coefficient (S_11_) and bandwidth (S_11_ < −10 dB), while also causing a slight shift in the resonant frequency. Therefore, an appropriate value of *g* should be selected to minimize S_11_ while ensuring the bandwidth fully covers the 77 GHz to 94 GHz band. Dimensional parameters are iteratively refined through 2D electromagnetic simulations to ensure the antenna meets operational requirements in both 2D and 3D configurations. After several iterations of 2D and 3D electromagnetic simulations, the final size specifications for the antenna are determined. The key geometrical parameters are summarized in Table 1.

**Table 1:** Antenna parameter size (Unit: μm)

Name	Size	Name	Size	Name	Size
*l*	15600	*w*	800	*d*	4000
*l*_d_	700	*w*_d_	300	*g*	100
*l*_f_	1775	*t*_Au_	0.3	*t*_PI_	5
*w*_f_	50	*s*_f_	50	*w*_p_	80
*s*_p_	125				

### Fabrication of the antenna

**Fig. 3:**
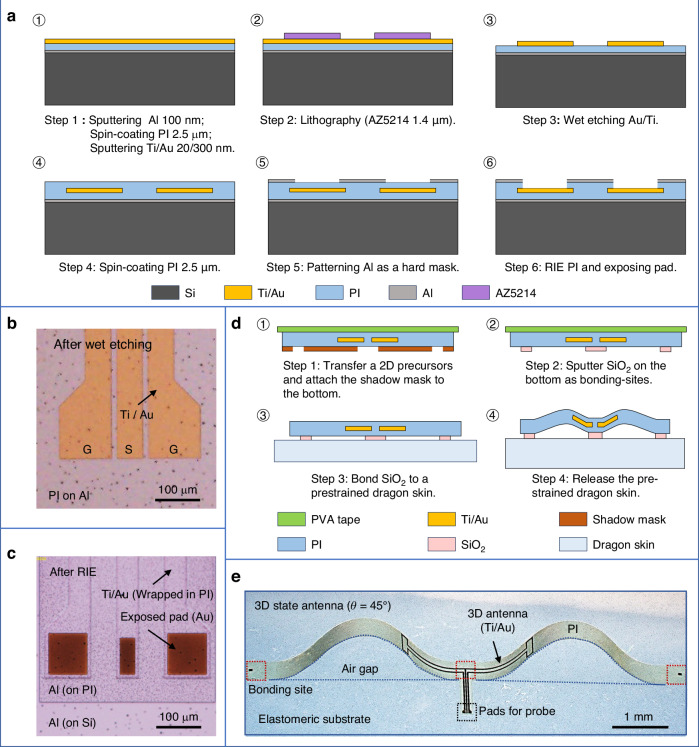
Schematic illustrations of the fabrication procedures for the antenna by MEMS technology and mechanically-guided method. **a** Schematic illustration of the microfabrication process for the 2D precursor. **b** Optical microscope image of the GSG pads on the 2D precursor following the wet etching process. **c** Optical microscope image of the metallic layers wrapped within the polyimide (PI) layer, with the GSG pads exposed via RIE for RF probe testing. **d** Schematic illustration of the transformation process that reshapes the 2D precursor into a 3D antenna by releasing the prestrained elastomeric substrate. **e** Optical microscope image demonstrating the stable 3D configuration achieved through prestrain release

A stepwise fabrication process for the proposed antenna is illustrated in Fig. 3. It comprises two main stages: the preparation of the 2D precursor and the buckling of the 3D antenna. The micro-electro-mechanical system (MEMS) fabrication technology is employed to prepare the 2D precursor, ensuring the manufacturing precision required for effective operation in the W-band. The mechanically-guided method is subsequently employed to achieve the 3D microarchitecture.

Briefly, as depicted in Fig. 3a, the microfabrication process of 2D precursor begins with the deposition of an aluminum (Al) sacrificial layer, a bottom polyimide (PI) layer, and a titanium/gold (Ti/Au) functional layer on a silicon (Si) wafer. After patterning metal layers via wet etching, a top PI layer is spin-coated, and both PI layers are defined using a reactive ion etching (RIE) process through a patterned Al hard mask. Figure 3b, c show the microscopic images of the antenna’s pads after wet etching and RIE, respectively. The precursor is then released using diluted hydrochloric acid and transferred onto polyvinyl alcohol (PVA) tape. Silicon dioxide (SiO_2_) bonding sites are sputtered via a PI shadow mask, as shown in Fig. 3d. Finally, after removing the tape and bonding the precursor onto a prestrained elastomeric substrate, the flexible structure is buckled into a 3D state by releasing the prestrain of the elastomeric substrate, as shown in Fig. 3e. Further fabrication details are provided in the “Materials and Methods” section.

### Compressive buckling of the antenna

**Fig. 4:**
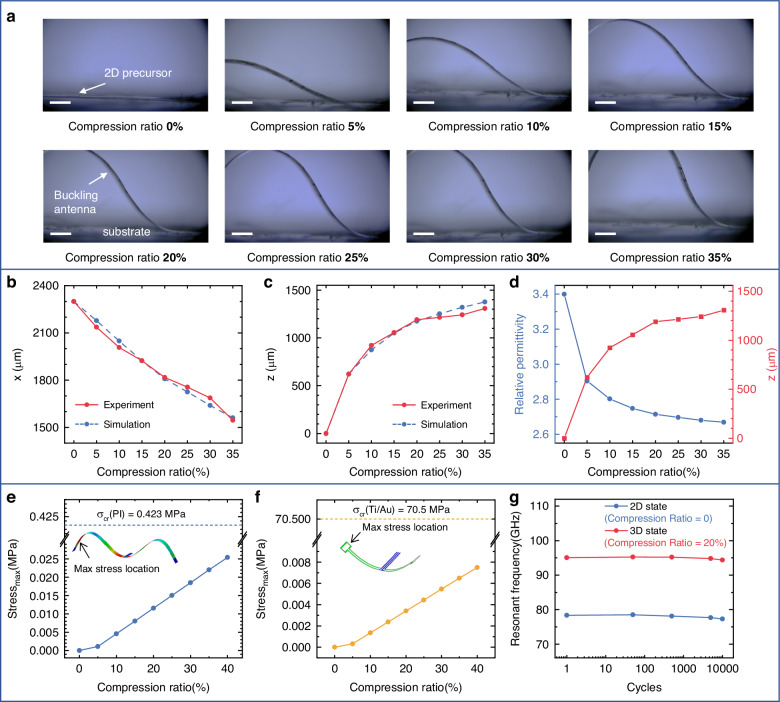
3D configurations of the antenna. **a** Optical images of the fabricated antenna under various compression ratios. Scale bar: 500 μm. **b**, **c** Simulated and measured positions of the radiating element’s extremity, where x represents the horizontal projection length of the antenna, and z denotes the height of the buckled antenna. **d** Variation in the calculated effective relative permittivity (*ε*_r,eff_) during the buckling process. Simulated stress during the buckling process for the PI film (**e**) and Ti/Au films (**f**). **g** Resonant frequencies after 1, 50, 500, 5,000, and 10,000 cycles of stretching and releasing

Quantitative mechanical modeling based on FEA predicts the 3D antenna configuration at various compression ratios, defined as (*l*_0_ - *l*_com_) / *l*_0_, where *l*_0_ represents the original length of the PI film, and *l*_com_ denotes the length of the PI film under compressive buckling. The optical images of the antenna’s cross-sectional profiles at different compression ratios, shown in Fig. 4a, closely align with the FEA predictions in Fig. 2b. Due to axial symmetry, both dipole ribbon components undergo identical deformations during the buckling process, each adopting a shape described by Eqs. (4) and (5)^47,48^:

4 $$h(x)=\frac{A}{2}\left(1+\,\cos \frac{4\pi (x-{l}_{{\rm{com}}}/4)}{{l}_{{\rm{com}}}}\right)$$

5 $$A=\frac{{l}_{0}}{\pi }\sqrt{\frac{{l}_{0}-{l}_{{\rm{com}}}}{{l}_{0}}-{\varepsilon }_{{\rm{c}}}}$$

where *h* represents the height of the PI ribbon, *A* denotes the maximum height of the PI ribbon and ${\varepsilon }_{{\rm{c}}}=\frac{4{\pi }^{2}{h}^{2}}{3{l}_{{\rm{com}}}^{2}}$ is the Euler buckling strain. As depicted in Fig. 4b, c, the measured locations (*x* and *z*) of the extremity of the radiating element are in accordance with the FEA simulation results. It is notable that the amount of lift (Δ*z*) gradually slows down. For instance, when the compression ratio increases from 0 to 5%, the Δ*z* is 621.875 μm, whereas increasing the compression ratio from 25% to 30% results in a minor change, with Δ*z* measuring 68.25 μm. According to Eqs. (2) (4) (5), and the measured height in Fig. 4 d, the calculated effective relative permittivity *ε*_r,eff_ between the antenna and the substrate decreases as the buckling height increases. Consequently, as the compression ratio varies within the range of 0% to 20%, the operating frequency of the antenna exhibits a continuous tunability, increasing from 77 GHz to 94 GHz. Beyond a 20% compression ratio, the antenna maintains stable performance. Furthermore, as shown in Fig. 4a, upon release of the substrate prestrain and the antenna’s transformation from a 2D to a 3D configuration, stretching the substrate allows the antenna to reversibly return to its 2D state, with the corresponding positions across compression ratios remaining unchanged.

The stress experienced by the 20/300 nm thick Ti/Au films wrapped in a 5 μm PI film during the transformation from 2D to 3D is analyzed using ABAQUS, and the locations experiencing the maximum stress are identified, as shown in Figs. S4, S5. As the compression ratio increases from 0% to 40%, the maximum stress gradually rises but remains significantly below the critical fracture stress (σ_cr_), as depicted in Fig. 4e, f. The maximum stress observed in the simulations is 0.0254 MPa for the PI film and 0.0075 MPa for the Ti/Au film, corresponding to 6.00% and 0.0106% of their respective critical fracture stresses. These results confirm a substantial safety margin, ensuring the materials’ mechanical integrity during the transformation process. Potential fatigue effects resulting from repeated mechanical deformations are also considered to ensure long-term reliability, as shown in Figs. 4g, S6. The antenna is tested under 50, 500, 5,000, and 10,000 cycles of loading and unloading at 40% compressive strain. The results indicate that the antenna maintains a stable resonant frequency during 10,000 cycles, with only minor shifts of approximately −1.35% in the 2D state and −0.726% in the 3D state under a 20% compression ratio. These shifts are minimal and within acceptable limits, highlighting the robustness of the design against fatigue failure of both the metal and the substrate.

### Electromagnetic performance of the antenna

**Fig. 5:**
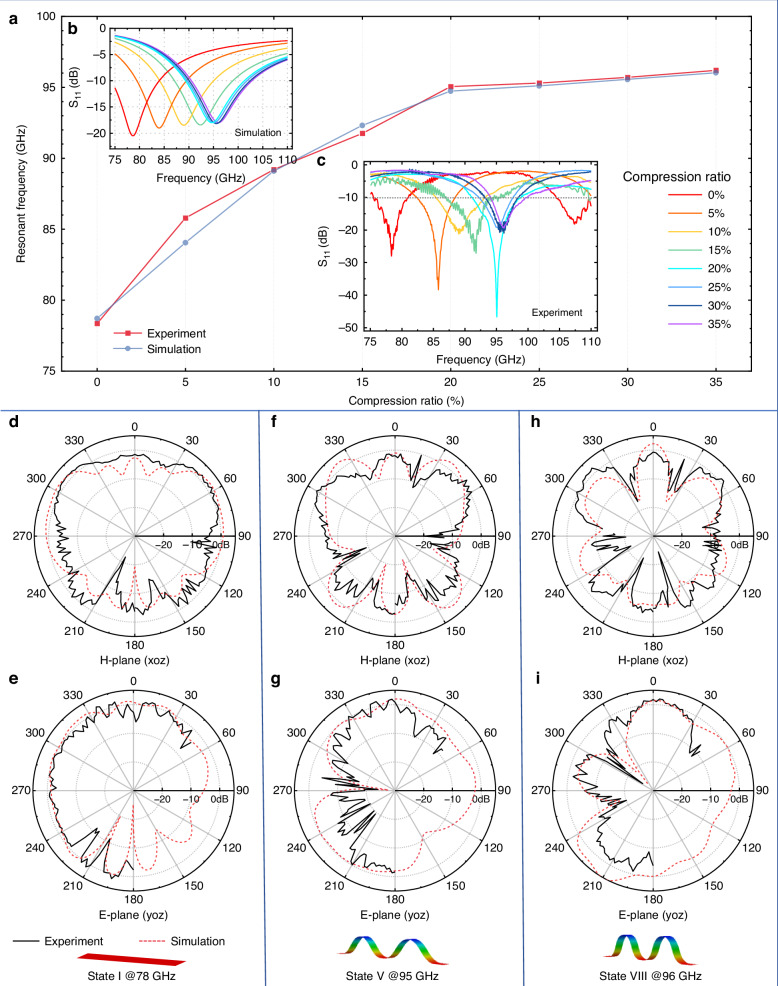
The electromagnetic performance of the mechanically-guided reconfigurable antenna at different states. **a** Simulated and experimental resonant frequencies of the buckling reconfigurable antenna at different states. **b** Simulated reflection coefficient (S_11_) characteristics of the antenna at eight different states. **c** The experimental S_11_ curve. **d**–**i** Radiation patterns of the antenna at different buckling states, illustrating performance for the 77 GHz and 94 GHz bands

The antenna is tested using a customized quasi-in-air-based far-field antenna measurement system at Shanghai Jiao Tong University^49^, as shown in Figure S7. The detailed test process is described in the “Materials and Methods” section. The simulated reflection coefficient (S_11_) curves in Fig. 5a indicate that the antenna can be tuned continuously from the 77 GHz band to the 94 GHz band, positively correlated with the compression ratios. Specifically, when the compression ratio exceeds 20%, the resonant frequency shows minor increments, suggesting the stability of the 3D antenna’s operating frequency. This slight level of variation is attributed to the reduced buckling deformation at high compression ratios.

The experimental results are measured by a vector network analyzer (VNA). By releasing the prestrained elastomeric substrate, the antenna exhibits different resonant frequencies corresponding to various compression ratios applied during testing. This capability allows for dynamic tuning of performance characteristics; specifically, stretching and releasing the elastomeric substrate enables continuous control of resonant frequency across a range from 77 GHz to 94 GHz. As the compression ratio increases, the buckling PI ribbon permits the antenna to operate in distinct frequency bands across eight states: State I (0% compression), State II (5%), State III (10%), State IV (15%), State V (20%), State VI (25%), State VII (30%), and State VIII (35%). These results demonstrate the reconfigurable antenna’s ability to accommodate a broad range levels of compression ratios, showcasing excellent frequency reconfigurability during the stretching and releasing process. The S_11_ curve exhibits an evident monotonous rightward shift from state I to state V, primarily attributed to a decrease in equivalent dielectric constant associated with buckling radiating elements as they undergo deformations. Mechanical modeling provides a reasonable explanation for these results. Specifically, the measured resonant frequencies, corresponding to the minimum S_11_ values, are as follows: 78.36 GHz (State I), 85.80 GHz (State II), 89.20 GHz (State III), 91.76 GHz (State IV), 95.05 GHz (State V), 95.30 GHz (State VI), 95.70 GHz (State VII), and 96.20 GHz (State VIII). Notably, as depicted in Fig. 5b, c, these experimental results closely align with simulated predictions, which are reported as follows: for respective states I through VIII, they are 78.71 GHz, 84.05 GHz, 89.10 GHz, 92.32 GHz, 94.73 GHz, 95.10 GHz, 95.56 GHz, and 96.03 GHz. The differences in minimum S_11_ values between simulated and experimental results are mainly attributed to GSG probe positioning. The main operating bands of the antenna can remain stable at the 77 GHz band as the 2D state and at the 94 GHz band as the 3D state (when the compression ratio exceeds 20%). In other words, to maintain the antenna operating at 94 GHz band, compression ratios larger than 20% are applicable, highlighting the flexibility and robustness of the strain required during operation. Additionally, the operational bandwidth (S_11_ < −10 dB) expands from 5.54 GHz (75.50–81.04 GHz) to 7.87 GHz (91.11–98.98 GHz). This expansion is attributed to the low relative permittivity and minimal loss within the air gaps formed during the buckling process.

The gain patterns of the antenna are tested by rotating the receiving horn antenna in the xoz and yoz planes. Due to blockage from the antenna under test (AUT) and the measurement platform, the horn antenna could only be rotated from −180° to +50° in the yoz plane. Figure 5d–i illustrate both the measured and simulated radiation patterns of the proposed antenna. In the main operating bands, the antenna achieves peak gains of 1.92 dBi at 78 GHz (state I), 1.91 dBi at 95 GHz (state VI), and 2.03 dBi at 96 GHz (state VIII). The measurements and simulations show good agreement, particularly around the main lobe, with the measured values being slightly lower than the simulated ones. The shape of the radiation patterns shows minor differences due to the buckling of the radiation elements, which alters the current distribution of the radiating elements. However, the primary radiation directions of the antenna remain consistent in the 3D state for 94 GHz band applications. Consequently, the frequency reconfigurable antenna, mounted on a flexible elastomeric substrate, can be readily deformed to accommodate automotive platforms that operate in the W band, possess complex curvilinear surfaces, and require multifunctionality from a single antenna.

## Discussion

To highlight the advantages of the proposed antenna employing the mechanically-guided method, a comparative analysis with other reconfigurable antennas is presented in Table 2. The existing literature contains relatively few studies on frequency reconfigurable antennas operating in the mm-wave frequency bands, particularly within the W-band. First, unlike antennas utilizing switchable components such as PIN diodes, varactors, and RF switches, the mechanically-guided method eliminates the need for bias circuits, minimizing static power consumption and reducing system complexity. Second, the proposed antenna achieves wideband and continuous tuning across the W-band, whereas most alternative methods are typically restricted to discrete operating states or limited bandwidths. Third, the normalized size of the proposed antenna is 1.4 × 10^−3^ λ_0_^3^, making it smaller than many existing designs in the mm-wave band. This compact form factor facilitates integration into lightweight and space-constrained systems. Finally, the flexibility of the antenna enables it to conform to complex surfaces, enhancing its versatility and applicability across diverse scenarios.

**Table 2:** Comparison of the proposed design with existing references

Ref.	Structure of Antenna	Reconfigurability Method	Number of Mode	Operating Bands (GHz)	Size (λ_0_^3^)^a^	Flexibility
^8^	Planar slot antenna	PIN-diode (bias voltage)	2	6.8, and 15.5	9.7 × 10^–3^	No
^12^	1×4 patch antenna	Varactor (bias voltage)	Continuous	23.2–30.2	0.023	No
^14^	2×2 Patch antenna	Varactor (bias voltage)	Continuous	4.65–5.42	9.6 × 10^–3^	No
^17^	Dipole-like design	Mechanically trigged switches	5	2.3–7.7	2.7 × 10^–4^	Yes
^19^	Patch antenna	Ni-Ti shape memory alloy	2	3.4–10.2, and 4.7–6.0	0.22	No
^21^	Metasurface antenna	Shape memory polymers	Continuous	11.3 to 13.5	0.016	Yes
^25^	Metasurface antenna	Phase change material VO_2_	2	23.25–24.3, and 37–39.8	0.16	No
^28^	Patch antenna	Thermistor (heat)	Continuous	1.85–2.3	6.1 × 10^–4^	Yes
^36^	Dipole antenna	Compressive buckling	Pattern reconfigurable	2.45 band	1.7 × 10^–4^	Yes
^37^	Dipole-like antenna	Compressive buckling	Pattern reconfigurable	2.45 band	1.4 × 10^–4^	Yes
**This work**	**Dipole-like antenna**	**Compressive buckling**	**Continuous**	**77–94**	**1.4** **×** **10**^**–3**^	**Yes**

^a^The dimensions are given in terms of λ_0_, the wavelength in free space at the center frequency of the tuning range

In summary, this work represents the first demonstration of achieving frequency reconfigurability in the W-band by compressive buckling. The antenna’s resonant frequency shifts dynamically from 78.35 GHz (with a 5.54 GHz bandwidth) to 95.06 GHz (with a 7.87 GHz bandwidth) by transitioning from a 2D to a 3D configuration through mechanical deformation, resulting in a frequency ratio of approximately 1:1.213. The multi-state antenna maintains consistent radiation patterns across different operating bands, with peak gains of 1.92 dBi at 78 GHz, 1.91 dBi at 95 GHz, and 2.03 dBi at 96 GHz. By integrating mechanical simulation and electromagnetic modeling, the antenna’s geometric configurations are meticulously optimized to achieve switchable RF performance. The 2D precursor, fabricated using MEMS technology, is assembled onto a prestrained elastomeric substrate and reshaped into a 3D configuration by compressive buckling. To validate the antenna’s robustness and long-term reliability, comprehensive fracture analysis confirmed that the stresses induced during the 2D-to-3D transformation remain well below critical fracture thresholds. Additionally, fatigue testing conducted over 10,000 deformation cycles under 40% compressive strain demonstrated stable resonant frequency performance, with only minor shifts of −1.35% in the 2D state and −0.726% in the 3D state. This exceptional reconfigurability and mechanical robustness position the antenna as a promising candidate for integrating into compact, multifunctional wireless systems, such as automotive platforms and automated logistics systems. Potential applications include advanced automotive radar, logistics robots, and automated guided vehicles (e.g., localization and obstacle detection at 77 GHz and high-resolution imaging and precise object tracking at 94 GHz).

## Materials and methods

### Selected antenna design

The dual-layer PI, with a dielectric constant of 3.9, sandwiches the Ti/Au radiating element. Both PI layers are 2.5 μm thick (*t*_PI_), while the Ti/Au layers are 20/300 nm thick (*t*_Au_). The length of the folded dipole antenna (*l*_d_) is determined to match a quarter of the guided wavelength (λ_g_/4), and the distance between the dual folded dipoles (*d*) is designed based on the free-space wavelength (λ_0_).

### Materials and manufacturing

The micro-electro-mechanical system (MEMS) fabrication technology was employed to prepare the 2D precursor, ensuring the manufacturing accuracy necessary for the antenna to function effectively in the W-band. The microfabrication process began with the deposition of an Al sacrificial layer (100 nm), a bottom polyimide (PI) layer (2.5 μm), and Ti/Au functional layers (20/300 nm) on a Si wafer. The antenna radiating elements were patterned using lithography and wet etching processes. A top PI (2.5 μm) layer was then spin-coated to protect the metallic films. Both the PI layers were patterned using a reactive ion etching (RIE) process through another Al (100 nm) hard mask to define the 2D precursor configuration and expose the ground-signal-ground (GSG) pads. The 3D microarchitecture was achieved using a mechanically-guided method. Then, the 2D precursor was released using diluted hydrochloric acid (HCl) and transferred onto a water-soluble polyvinyl alcohol (PVA) tape. A laser-cut PI shadow mask was aligned to expose the bonding sites with the 2D precursor side down. After sputtering SiO_2_ (50 nm) through the shadow mask, the precursor was bonded onto a prestrained elastomeric substrate (Dragon Skin 10 slow, Smooth-On). Chemical covalent bonds between the SiO_2_ and the elastomeric substrate were yielded in an oven at 60 °C for 20 min. The PVA tape was then dissolved and rinsed away with water. Finally, the flexible structure was buckled into a 3D state by releasing the prestrain of the elastomeric substrate.

### Simulation of the mechanical performance

The commercial software Abaqus was utilized to allow the mechanical simulations to predict the 3D configuration of the antenna. The four-node 2D elements with PI-Ti/Au-PI laminates were adopted to model the 2D precursors, and eight-node 3D solid elements (C3D8R) were adopted to model the elastomeric substrate. The critical buckling strains and corresponding buckling modes determined from linear buckling analyses were implemented as initial imperfections in the post-buckling calculations to obtain the deformed 3D configurations and strain distributions. The elastic modulus (E) and Poisson’s ratio (ν) are E_substrate_ = 166 kPa and ν_substrate_ = 0.49 for substrate (Dragon Skin, Smooth-On); E_Ti/Au_ = 78 GPa and *ν*_Ti/Au_ = 0.44 for Ti/Au; and E_PI_ = 2.5 GPa and *ν*_PI_ = 0.34 for PI.

### Simulation of the electromagnetic performance

The commercial software HFSS was utilized to allow the electromagnetic simulations to calculate the coefficient (S_11_), impedance (Z_11_), and radiation pattern of the antenna. Convergence of mesh sizes was tested to ensure computational accuracy. The 3D geometries of the antenna under different compression ratios obtained by the mechanic simulations were imported to the HFSS. All the metal conducting layers and polymeric supporting layers were defined with prescribed thicknesses. The relative permittivity (ε_r_), relative permeability (μ_r_), and conductivity (σ) are σ_Au_ = 5.8 × 10^7 ^S/m; *ε*_r_PI_ = 3.9, *μ*_r_PI_ = 1, and *σ*_PI_ = 0 S/m; and *ε*_r_Substrate_ = 3.4, *μ*_r_Substrate_ = 1, and *σ*_Substrate_ = 2.5 × 10^−14^ S/m.

### Optimization of the antenna design

The optimization procedure for the frequency reconfigurable antenna design involves the following steps: (a) Initial Material and Geometric Selection: Low relative permittivity materials (PI and Dragon Skin) are selected based on the design requirements. Electromagnetic simulations are conducted on the 2D precursor structure to establish the fundamental parameters of the antenna at 77 GHz, including the length of the dipole antenna. (b) 3D Transformation and Modeling: The optimized 2D precursor structure is transformed into a 3D configuration using ABAQUS. The resulting structures are input into electromagnetic simulation software for 3D antenna simulations. (c) 3D Antenna Adjustment: The length of the 3D antenna is fine-tuned iteratively to adjust the resonant frequency to 94 GHz. The spacing between strips is optimized to minimize S_11_ at 94 GHz while ensuring adequate bandwidth coverage. (d) Feedback and Refinement: Key design parameters obtained from the 3D simulations are fed back into the 2D precursor model to refine its electromagnetic properties. Steps (a) through (c) are repeated iteratively until the desired metrics are achieved.

### Measurement of the resonant frequency and gain

The antenna was tested using a quasi-in-air-based far-field antenna measurement system settled in a 3.5 m × 3.5 m × 2.8 m microwave anechoic chamber customized by Shanghai Jiao Tong University and can test antennas fed by a waveguide or a probe from 18 to 325 GHz. The measurement setup involves an antenna-under-test (AUT) transmitting and a standard horn antenna with a mixer receiving, and the AUT is fed by an Infinity probe I110-GSG-150-BT with a pitch of 150 μm. The horn antenna is mounted on a rotating arm, establishing a 60 cm spherical far-field condition between the AUT and the horn antenna, allowing the measurement system to test an antenna’s input impedance and gain pattern of an antenna.

## Supplementary information

Supplemental Material
