# Supplementary material for: A stretchable frequency reconfigurable antenna controlled by compressive buckling for W-band applications
Source: Microsyst Nanoeng. 2025 May 13;11:86. doi: 10.1038/s41378-025-00890-x (PMC12075831; doi:10.1038/s41378-025-00890-x)
Supplement: Supplementary file 1 — Supplemental Material [file 41378_2025_890_MOESM1_ESM.docx]

SUPPLEMENTARY MATERIALS

A Stretchable Frequency Reconfigurable Antenna Controlled by Compressive Buckling for W-Band Applications

Qi Wang^1,2^, Zetian Wang^2^, Yang Yang^1,2^, Chi Zhang^2,3,4^, Mengdi Han^5*^, Wei Wang^2,3,4*^, and Yufeng Jin^1,2,3*^

^1^ Peking University Shenzhen Graduate School, Peking University, Shenzhen, China

^2^ School of Integrated Circuits, Peking University, Beijing, China

^3^ National Key Laboratory of Advanced Micro and Nano Manufacture Technology, Beijing, China

^4^ Beijing Advanced Innovation Center for Integrated Circuits, Beijing, China

^5^ School of Future Technology, Peking University, Beijing, China

*: corresponding authors.

### Corresponding authors

Mengdi Han, [hmd@pku.edu.cn](mailto:hmd@pku.edu.cn);

Wei Wang, [w.wang@pku.edu.cn](mailto:w.wang@pku.edu.cn);

Yufeng Jin, [yfjin@pku.edu.cn](mailto:yfjin@pku.edu.cn)


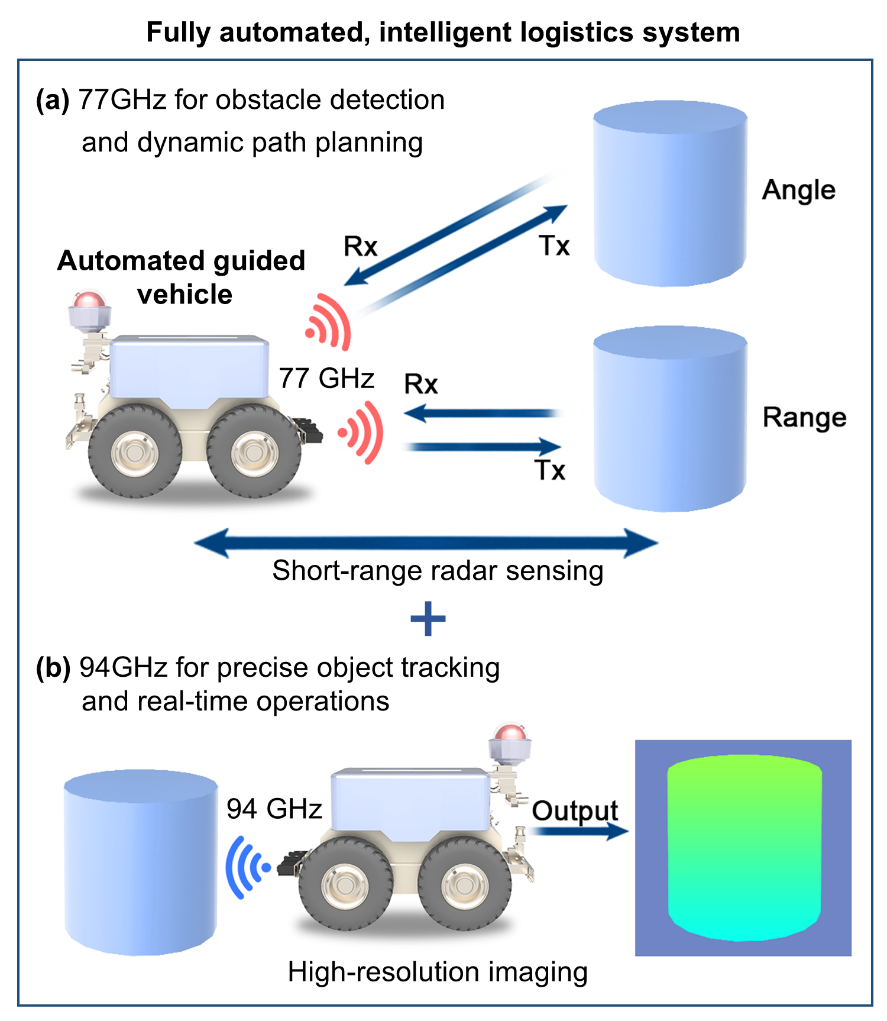


**Figure S1** Potential practical applications of the proposed antenna in fully automated and intelligent logistic systems, including **(a)** localization in the 77 GHz band for obstacle detection and dynamic path planning, and **(b)** high-resolution imaging in the 94 GHz band for precise object tracking and real-time operational tasks, such as sorting, loading, and unloading.


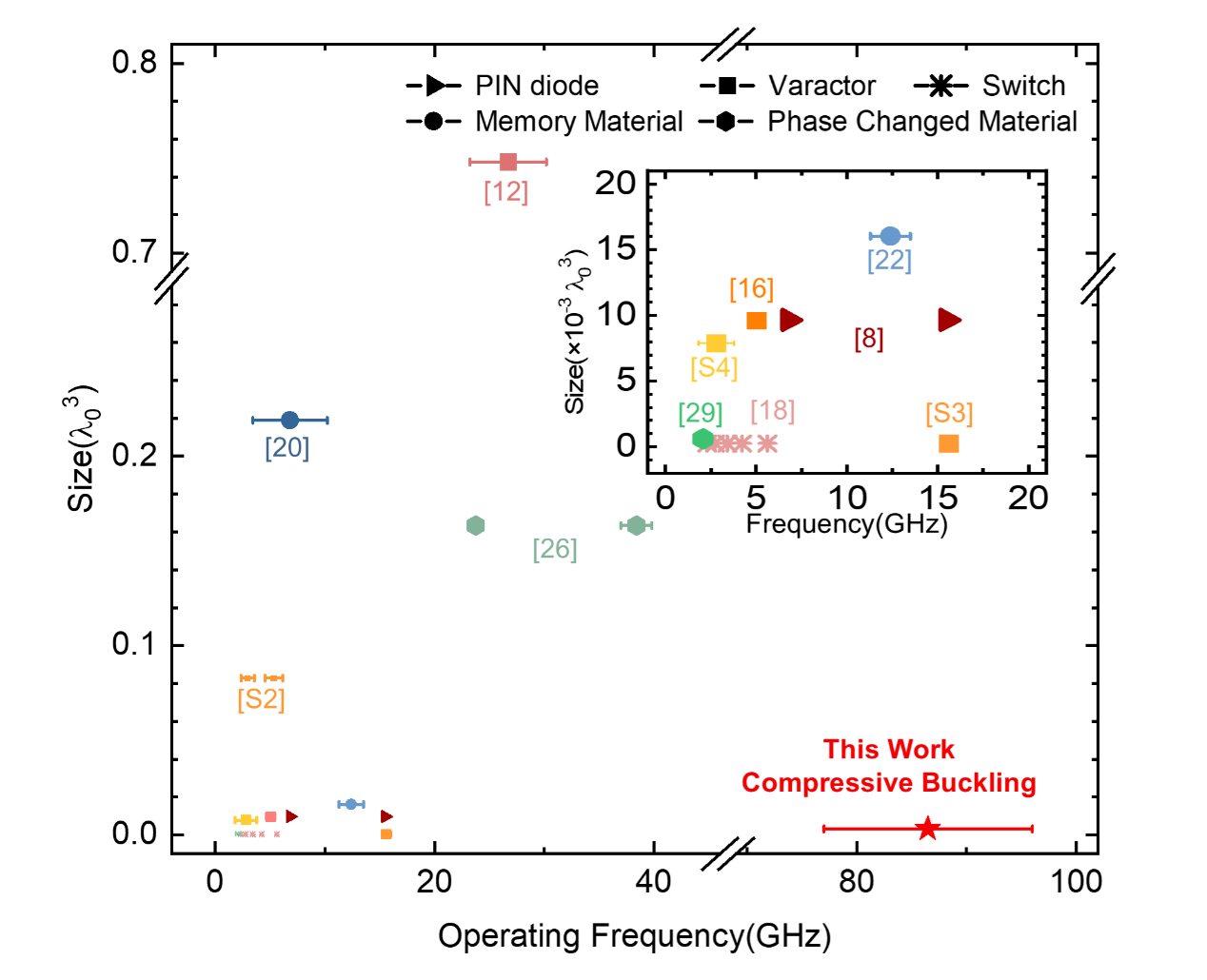


**Figure S2** Reconfigurable antenna performance comparison chart, where the lines represent the operating bandwidth of the antennas. The proposed antenna offers key advantages such as miniaturization and a broader tunable bandwidth compared to other designs. The dimensions are given in terms of λ_0_, the wavelength in free space at the center frequency of the tuning range.


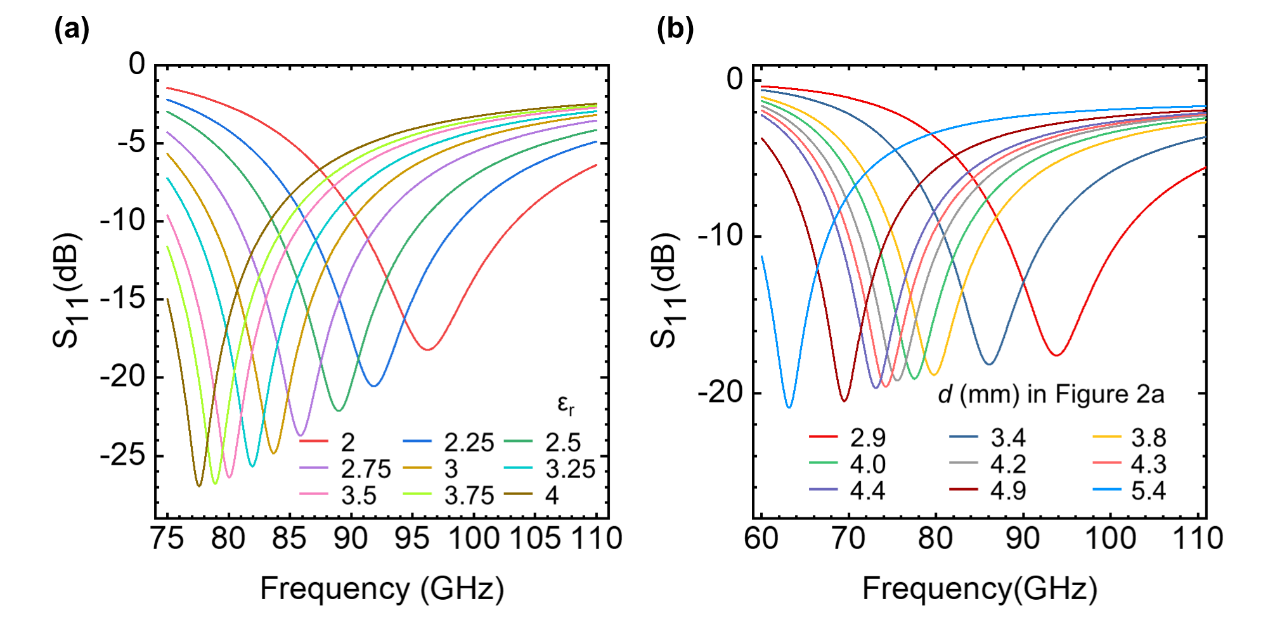


**Figure S3** Electromagnetic simulation results for key parameters of the antenna. **(a)** S_11_ characteristics of varying substrate relative permittivity (*ε*_r_). **(b)** S_11_ characteristics of varying length (*d*) of the proposed antenna.


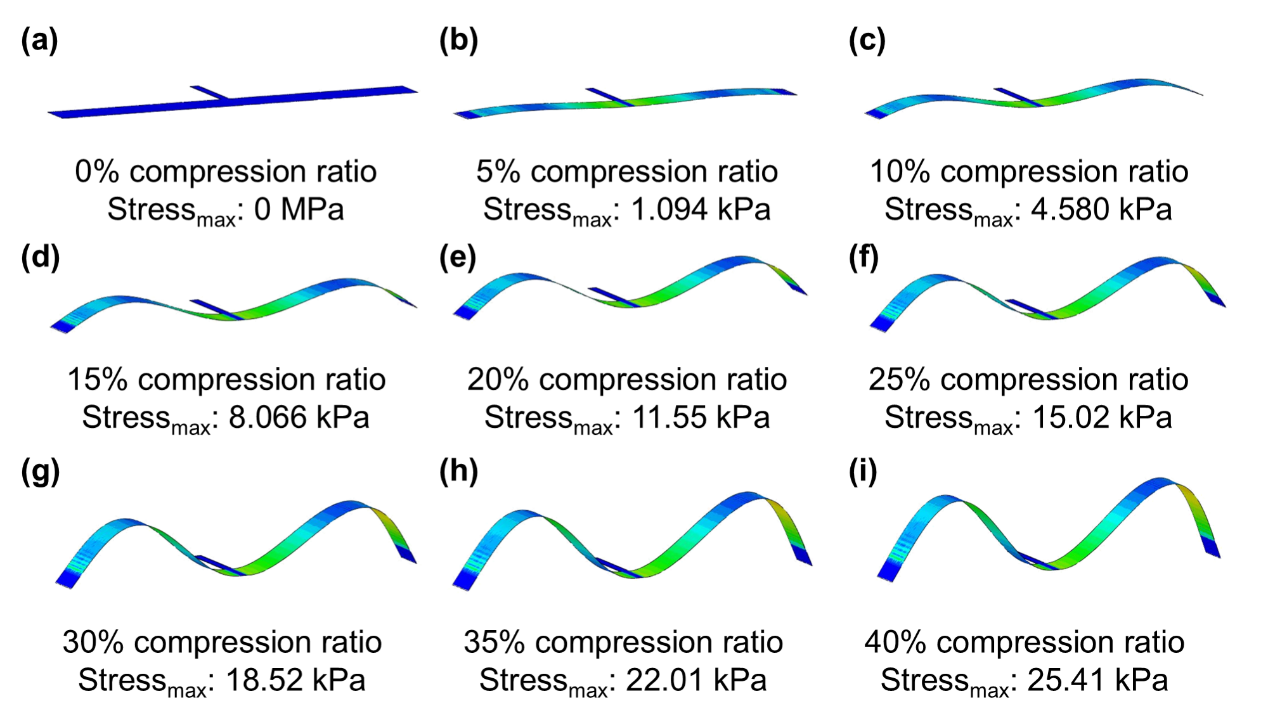


**Figure S4** Mechanical simulation results of the PI film fracture during the transformation from a 2D precursor to a 3D structure.


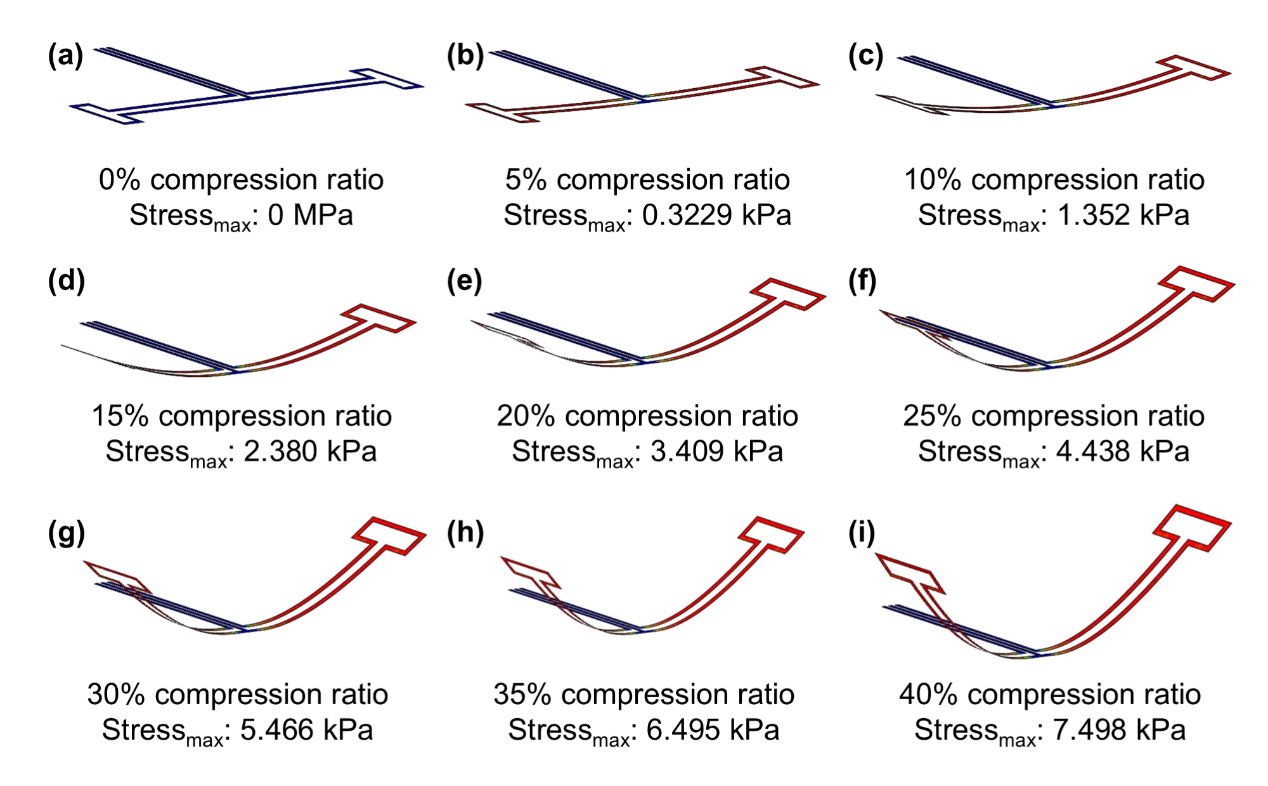


**Figure S5** Mechanical simulation results of the Ti/Au film fracture during the transformation from a 2D precursor to a 3D structure.


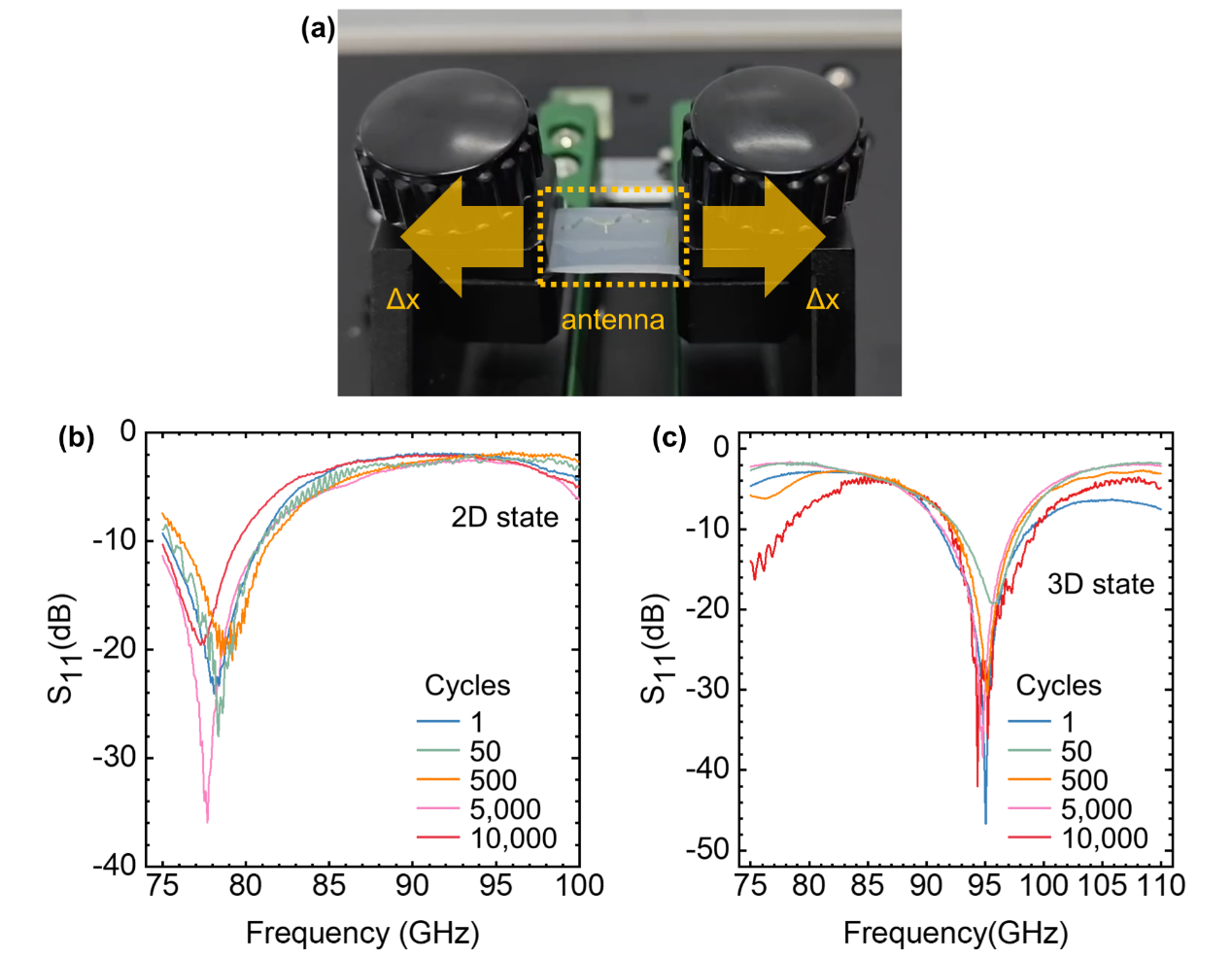


**Figure S6** Fatigue failure test results for metals or substrates subjected to repeated mechanical deformations. **(a)** An image of the cyclic tensile testing setup utilized in the fatigue test. **(b - c)** S_11_ characteristics of the antenna after undergoing multiple cycles in its **(b)** 2D state and **(c)** 3D state with a compression ratio of 20%.


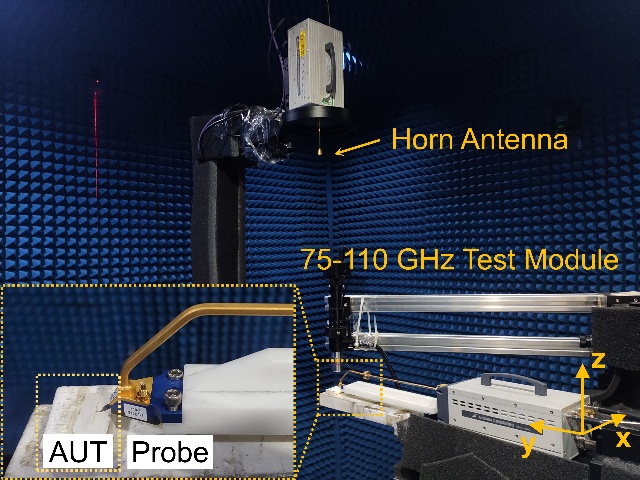


**Figure S7** Electromagnetic performance measurement setup, involves an antenna-under-test (AUT) transmitting and a standard horn antenna with a mixer receiving. The horn antenna is mounted on a rotating arm, establishing a 60 cm spherical far-field condition between the AUT and the horn antenna. This configuration enables the system to measure the reflection coefficients and gain patterns of the antenna.

**Table S1 Electrical Properties of Ti/Au Film at 77 GHz and 94 GHz**

| **Frequency (GHz)** | **Conductivity (×10^6^S/m)** | **Skin depth (μm)** |
| --- | --- | --- |
| **77** | **45.2** | **0.2715** |
| **94** | **45.2** | **0.2457** |

The skin depth is calculated by the Equation (ES1),

$\delta=\sqrt{\frac{1}{\pi\sigma\mu f}}$ (ES1)

where *σ*, *μ*, and *f* are conductivity, permeability, and frequency, respectively.

**Table S2 Critical Fracture Stresses (σ_cr_) of PI and Ti/Au Films.**

| **Material** | **Thickness (nm)** | **Kc (MPa·**$\sqrt{\mathbf{m}}$**)** | **σ_cr_ (MPa)** |
| --- | --- | --- | --- |
| PI | 5,000 | 1.5 | 0.423 |
| Ti/Au | 20/300 | 25 | 70.5 |

The σ_cr_ is calculated using Linear Elastic Fracture Mechanics (LEFM) [S1]:

$\sigma_{\text{rc}}=\frac{K_{\text{c}}}{Y\sqrt{\pi a}}$ (ES2)

where *K*_c_ is the fracture toughness (1.5 MPa·$\sqrt{m}$ for the PI film, and 25 MPa·$\sqrt{m}$ for the Ti/Au film), *Y* is the geometry correction factor, and *a* is the crack length. The calculated results are obtained using *a* = 10 nm and *Y* = 2.

**Table S3 Comparison of the Proposed Design with Existing References**

| **Ref.** | **Structure of Antenna** | **Reconfigurability Method** | **Number of Mode** | **Operating Bands (GHz)** | **Size (λ_0_^3^)^*^** | **Flexibility** |
| --- | --- | --- | --- | --- | --- | --- |
| [S2] | Patch antenna | Varactor | Continuous | 2.34 to 3.55, and 4.55 to 6.15 | 0.083 | Yes |
| [S3] | Slot-loop antenna | ferroelectric  K_0.5_Na_0.5_NbO_3_ (KNN) varactors | 2 | 15.22 to 15.97 | 2.5×10^-4^ | No |
| [S4] | Patch antenna | Varactor | Continuous | 1.82 to 3.77 | 7.9×10^-3^ | Yes |
| **This work** | **Dipole-like antenna** | **Mechanically-guided method** | **Continuous** | **77 to 94** | **1.4×10^-3^** | **Yes** |

*The dimensions are given in terms of λ_0_, the wavelength in free space at the center frequency of the tuning range.

**References**

[S1] T. L. Anderson, *Fracture Mechanics: Fundamentals and Applications, Fourth Edition*, 4th ed. Boca Raton: CRC Press, 2017, doi: 10.1201/9781315370293.

[S2] Q. H. Dang, S. J. Chen, D. C. Ranasinghe, and C. Fumeaux, “Dual-Band Reconfigurable Flexible Antenna With Independent Frequency Tunability,” *Antennas Wirel. Propag. Lett.*, vol. 22, no. 3, pp. 531–535, Mar. 2023, doi: 10.1109/LAWP.2022.3217256.

[S2] B. Aspe *et al.*, “Frequency-Tunable Slot-Loop Antenna Based on KNN Ferroelectric Interdigitated Varactors,” *Antennas Wirel. Propag. Lett.*, vol. 20, no. 8, pp. 1414–1418, Aug. 2021, doi: 10.1109/LAWP.2021.3084320.

[S4] Q. H. Dang, S. J. Chen, D. C. Ranasinghe, and C. Fumeaux, “A Frequency-Reconfigurable Wearable Textile Antenna With One-Octave Tuning Range,” *IEEE Trans. Antennas Propagat.*, vol. 69, no. 12, pp. 8080–8089, Dec. 2021, doi: 10.1109/TAP.2021.3083826.
